# The Occurrence of Seedlessness in Higher Plants; Insights on Roles and Mechanisms of Parthenocarpy

**DOI:** 10.3389/fpls.2018.01997

**Published:** 2019-01-18

**Authors:** Maurizio E. Picarella, Andrea Mazzucato

**Affiliations:** Laboratory of Biotechnologies of Vegetable Crops, Department of Agriculture and Forest Sciences, University of Tuscia, Viterbo, Italy

**Keywords:** adaptation, apomixis, inventory, parthenocarpy, *Solanum lycopersicum*, tomato

## Abstract

Parthenocarpy in a broad sense includes those processes that allow the production of seedless fruits. Such fruits are favorable to growers, because they are set independently of successful pollination, and to processors and consumers, because they are easier to deal with and to eat. Seedless fruits however represent a biological paradox because they do not contribute to offspring production. In this work, the occurrence of parthenocarpy in Angiosperms was investigated by conducting a bibliographic survey. We distinguished monospermic (single seeded) from plurispermic (multiseeded) species and wild from cultivated taxa. Out of 96 seedless taxa, 66% belonged to plurispermic species. Of these, cultivated species were represented six times higher than wild species, suggesting a selective pressure for parthenocarpy during domestication and breeding. In monospermic taxa, wild and cultivated species were similarly represented. The occurrence of parthenocarpy in wild species suggests that seedlessness may have an adaptive role. In monospermic species, seedless fruits are proposed to reduce seed predation through deceptive mechanisms. In plurispermic fruit species, parthenocarpy may exert an adaptive advantage under suboptimal pollination regimes, when too few embryos are formed to support fruit growth. In this situation, parthenocarpy offers the opportunity to accomplish the production and dispersal of few seeds, thus representing a selective advantage. Approximately 20 sources of seedlessness have been described in tomato. Excluding the EMS induced mutation *parthenocarpic fruit* (*pat*), the parthenocarpic phenotype always emerged in biparental populations derived from wide crosses between cultivated tomato and wild relatives. Following a theory postulated for apomictic species, we argument that wide hybridization could also be the force driving parthenocarpy, following the disruption of synchrony in time and space of reproductive developmental events, from sporogenesis to fruit development. The high occurrence of polyploidy among parthenocarpic species supported this suggestion. Other commonalities between apomixis and parthenocarpy emerged from genetic and molecular studies of the two phenomena. Such insights may improve the understanding of the mechanisms underlying these two reproductive variants of great importance to modern breeding.

## Introduction

“That some plants produce fruits without seeds is a fact observed and recorded by the ancients, according to Sturtevant in 1890” is the introductory statement reported in Gustafson's comprehensive work regarding the subject of parthenocarpy (Gustafson, 1942). The reasons for such an interest are soon after explained “because seedless fruits were thought to be better and also because many varieties are self-sterile, necessitating the planting of more than one variety in an orchard to insure a profitable crop” (Gustafson, 1942).

The production of seedless fruits (apireny or parthenocarpy *sensu lato*) has attracted since long time the farmers, because they are set independently of successful pollination. In addition, seedless fruits are favorable to processors, being their manipulation easier, and to consumers, being more pleasant to eat. Seedless fruits can occur when the ovary develops directly without fertilization (parthenocarpy *sensu stricto*) or when pollination and fertilization trigger ovary development, but the ovule/embryo aborts without producing mature seed (stenospermocarpy). The term parthenocarpy is hereby used in its broad sense to indicate both forms of apireny. Parthenocarpy is generally driven by genetic factors; nonetheless, seedlessness can be also induced with the application of various hormones to young inflorescences (Nitsch, 1952; Schwabe and Mills, 1981). Sources of genetic parthenocarpy are either obligate or facultative. In sexually propagated species, parthenocarpic genotypes should be facultative in order to be multiplied in conditions where the trait expressivity is lower. Differently, obligate parthenocarpy can be adopted in vegetatively propagated crops (Gorguet et al., 2005). From the adaptive point of view, the production of seedless fruits is an intriguing phenomenon, because empty fruits are costly to the mother plant and do not contribute to the production of offspring. When seed set fails, the abscission of the flower is the standard pathway to avoid the waste of resources in growing structures not fulfilling a biological purpose. The occurrence and permanence of parthenocarpy in plant populations is largely the effect of human selection that harnessed seedlessness as a commodity in fruit crops (Varoquaux et al., 2000). However, parthenocarpic genotypes are also found in wild species or in crops were the main product is not the fruit (non-fruit crops), indicating the possibility of adaptive reasons underlying empty fruit formation in higher plants.

In parallel with parthenocarpy that involves carpel development independent of pollination, the term parthenogenesis is used to indicate the development of an embryo in absence of male contribution. Parthenogenesis is part of the process called apomixis, a modified mode of reproduction resulting in seed production by asexual means (agamospermy, Nogler, 1984). Seeds of apomictic origin replicate the exact genome of the mother plant as they result from the parthenogenetic development of unreduced (apomeiotic) egg cells. In gametophytic apomixis, the apomeiotic egg cell is differentiated within an unreduced female gametophyte developing when a somatic nucellar cell acquires the developmental program of a megaspore (apospory) or when the meiocyte bypasses meiosis and proceeds directly with the gametophytic development (diplospory). In all cases, apomixis opens the possibility for cloning genotypes by seed. By consequence, harnessing apomixis is an exciting perspective for plant breeders and efforts to decipher its genetic control have been strongly pursued in the last decades (Albertini et al., 2010).

In this work, we present a bibliographic investigation of the occurrence of seedlessness within flowering plants and review hypothesis into the possible “adaptive” roles for parthenocarpy. To follow a case study, the inventory of the sources of parthenocarpy reported in tomato indicated that wide hybridization is involved in the majority of lines showing seedlessness in this species. Parallelisms with studies on apomicts offered novel cues into the mechanisms controlling parthenocarpy in angiosperms.

## Methods

Search of species where the occurrence of parthenocarpy has been described was carried out through the available literature. The main bibliographic source was the comprehensive report by Gustafson (1942) that was combined with other publications. For each species, the phylogenetic position was listed, according to the flowering plant classification of the Angiosperm Phylogeny Group III (APG III, 2009). The ovules/ovary (seeds/fruit) ratio was considered to distinguish the species in seed categories as “monospermic” (a single seed per fruit) and “plurispermic” (more-than-one-seed per fruit). In addition, species were distinguished according to their occurrence as wild or cultivated, and among the latter between fruit and non-fruit crops (species predominantly grown for the consumption of vegetative parts or for ornamental means). Finally, parthenocarpic species were classified according to their life form, fruit type, sex distribution and occurrence of polyploidy. Differences between distribution of diploid and polyploid species within classes of seed category, life form, sex distribution and status as crop or wild were estimated by χ^2^-test of 2 × 2 or 2 × 3 contingency tables.

The inventory of the sources of parthenocarpy described in tomato was carried out using a similar procedure. A first screening was based on the most comprehensive reviews (Philouze, 1983; George et al., 1984; Lukyanenko, 1991), and further details were found in additional publications, newsletters and bulletins.

To compare gene expression patterns, genes involved in fruit set in tomato were selected from the analysis reported on the *pat* mutant (Ruiu et al., 2015). We addressed those genes that are up-regulated after anthesis in the WT but not in the mutant (referred to as Pollination-dependent, PD group in Ruiu et al., 2015) and those that are up-regulated after anthesis in the mutant but not in the WT (referred to as Fruit growth-related, FG group). PD and FG gene lists have been used to retrieve gene expression at anthesis and few days after in cultivated tomato (*Solanum lycopersicum* L., formerly *Lycopersicon esculentum* Miller; cv M82) and in *S. pimpinellifolium* L. (formerly *L. pimpinellifolium* Miller) using the Tomato Expression Atlas (TEA) at the Sol Genomics Network website (sgn, https://solgenomics.net; Shinozaki et al., 2018). Comparable data were available for tissue-specific analysis on pericarp, placenta and septum. The ratio of expression after anthesis and at anthesis was calculated and expressed as logFC. Genes showing no expression after anthesis in TEA databases were discarded from the analysis, whereas genes showing no expression before anthesis were assigned the arbitrary value of 0.01 in order to allow a logFC value to be calculated. For the two groups of genes and the three tissues, the correlation coefficient between the logFC in Chico III and M82 and between Chico III and *S. pimpinellifolium* were calculated using SAS software package (SAS® University Edition).

## Results and Discussion

### Distribution of Parthenocarpy in Flowering Plants

**Figure 1:**
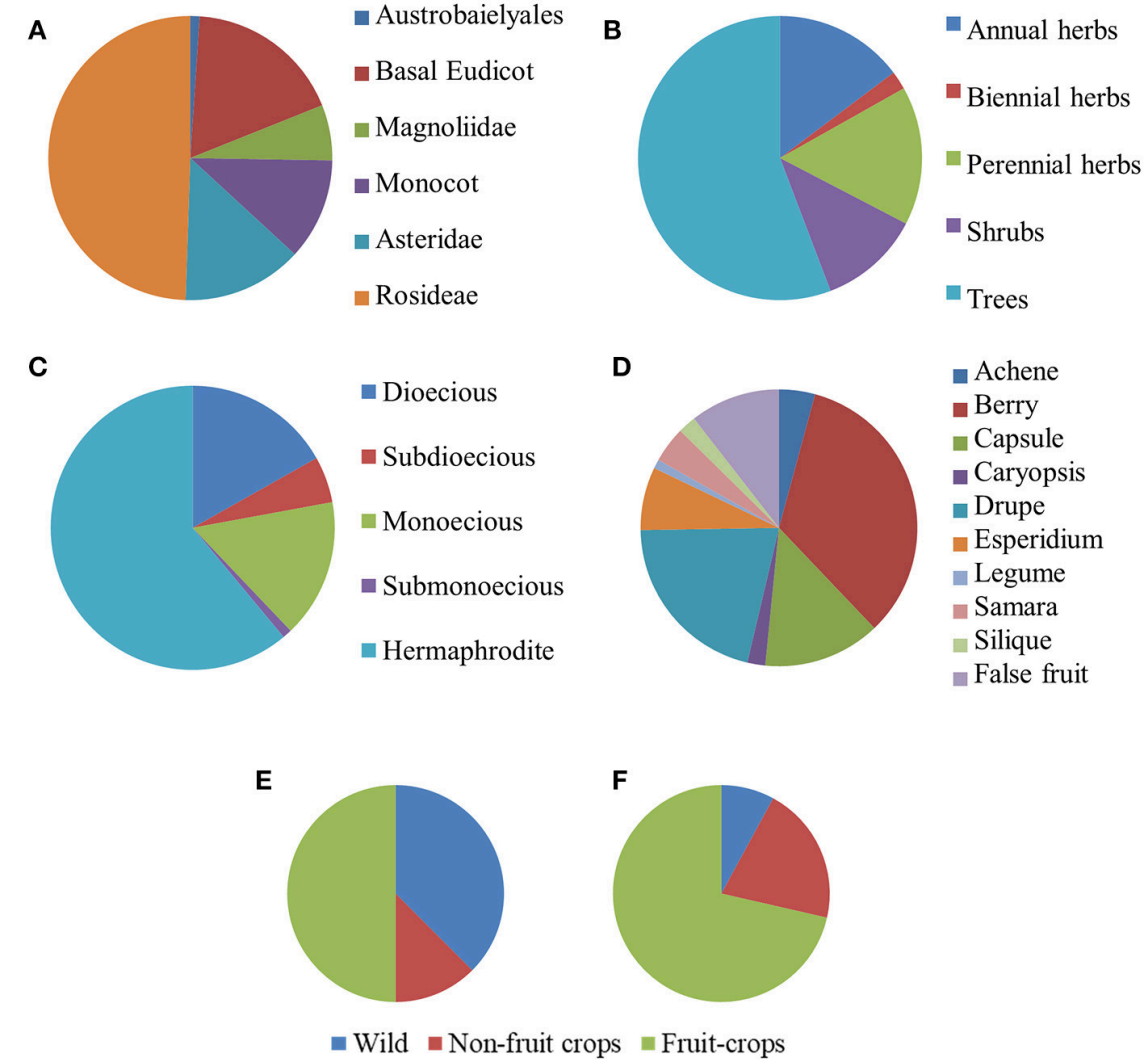
Distribution of species reported in literature for the occurrence of parthenocarpy (n = 95). Taxa are grouped according to the phylogenetic group **(A)**, life form **(B)**, sex distribution **(C)**, and fruit type **(D)**. Distribution of monospermic [**(E)**, *n* = 32] and of plurispermic [**(F)**, *n* = 63] species according to the status as wild, non-fruit crops and fruit crops.

After our search, parthenocarpy was reported in 96 Angiosperm taxa, 60 of which were listed in the (Gustafson, 1942) and the others were integrated from more recent sources (Table S1). Exactly one third of the species was classified as monospermic and the rest as plurispermic (Table S1). The most represented taxonomic group was the *Rosidae* (49.8%, Figure 1A), with a higher contribution of *Anacardiaceae* and *Rutaceae* (eight species each), *Rosaceae* (six species), and *Moraceae* (four species). The *Asteridae* contributed 13.8% of the species, with the strong prevalence of Solanaceae (nine species). Monocots were also present in the list (11.6%; Figure 1A). Notably, 17.9% of the listed species belonged to Basal Eudicots, where the Cucurbitaceae were prevalent (four species; Table S1).

**Figure 2:**
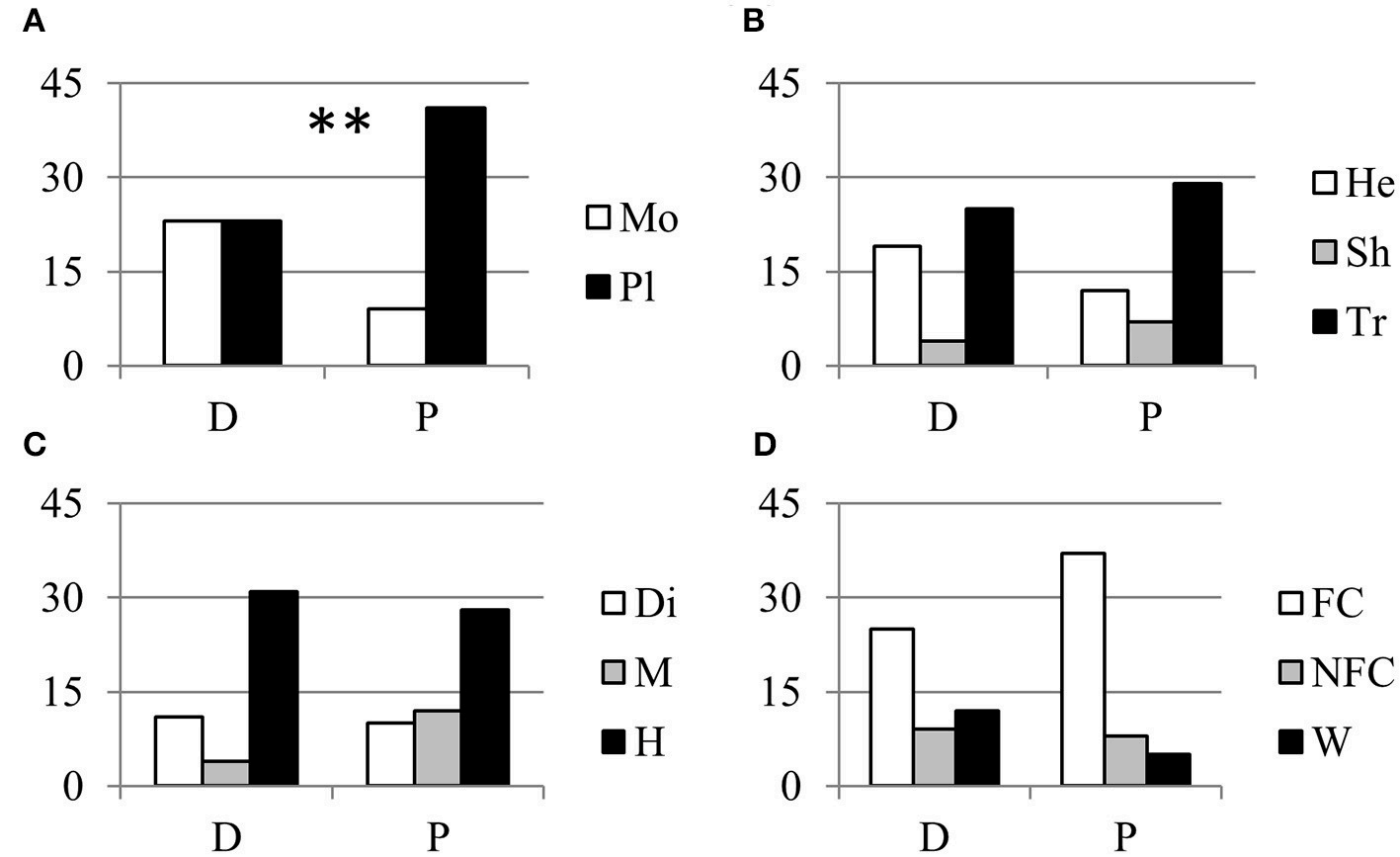
Relationship between parthenocarpy and polyploidy. Distribution of diploid (D) and polypoid (P) parthenocarpic species in relation with **(A)** the seed category (Mo, monospermic; Pl, plurispermic), **(B)** the life form (He, annual and perennial herbs; Sh, shrubs; Tr, trees), **(C)** the distribution of sexes (Di, dioecious; M, monoecious; H, hermafrodite) and **(D)** the status as fruit crop (FC), non-fruit crop (NFC), or wild species (W). ^**^ indicate distributions significantly different for *P* ≤ 0.01 after χ^2^-test of 2 × 2 or 2 × 3 contingency tables.

About one half of the parthenocarpic species were trees; the rest were equally distributed as annuals herbs, perennial herbs and shrubs (Figure 1B). More than one half of the species were hermaphrodite, whereas about 40% showed a form of sex separation (Figure 1C). Among species with monospermic fruits, the majority had a drupe as fruit type; among the plurispermics the most common fruit was the berry (Figure 1D). In monospermics, about 38% of species were wild and 50% were fruit-crops (Figure 1E). In plurispermic species, fruit-crops were predominant (71%), but still about 8% were wild and 21% were species cultivated, but not for products of the reproductive system (Figure 1F). About half of the parthenocarpic species (52.1%) were polyploid or showed instances of polyploidy or evidence of hybrid origin (Table S1). This frequency was significantly higher (χ^2^ = 13.1, *P* ≤ 0.01) than the general incidence of polyploidy in angiosperms (34.5%, Wood et al., 2009). Considering the above described classification of parthenocarpic species, polyploidy was unevenly distributed between monospermics and plurispermics, with a higher frequency in plurispermic species (Figure 2A). Differently diploid and polyploid species were evenly found among classes related to life form, to sex distribution or to the status as wild, non-fruit and fruit crops (Figures 2B–D).

**Figure 3:**
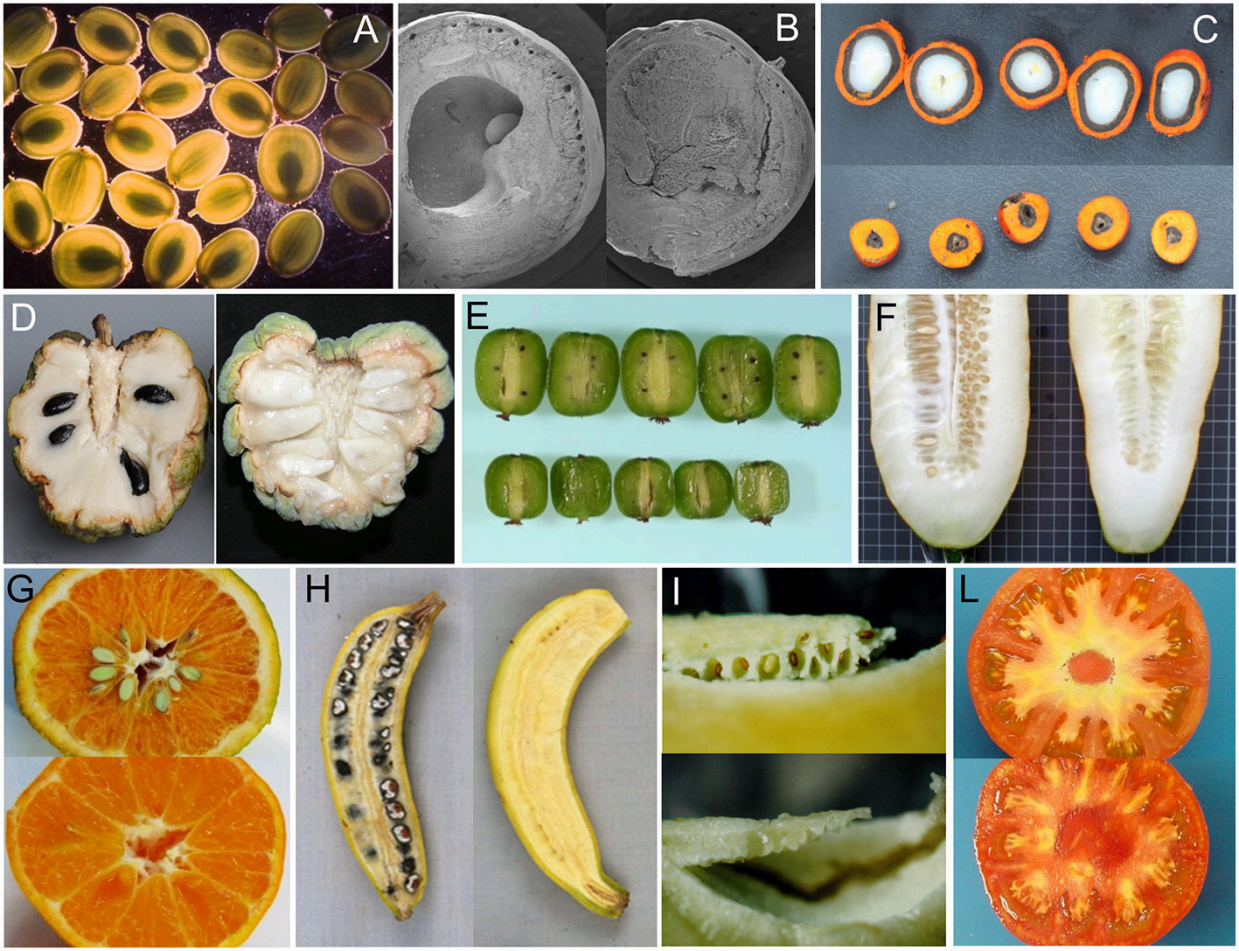
Examples of seeded and seedless fruits in parthenocarpic species. **(A)** *Pastinaca sativa* (courtesy of M.R. Berenbaum), **(B)** *Bursera aptera* (courtesy of M.F. Ramos Ordoñez), **(C)** *Elaeis oleifera* (courtesy of E. Barcelos), **(D)** *Annona squamosa* (from Lora et al., 2011), **(E)** *Actinidia arguta* cv Issai (courtesy of I. Kataoka), **(F)** *Cucumis sativus* (courtesy of M. J. Dìez), **(G)** *Citrus clementine* (courtesy of C. Mesejo), **(H)** *Musa acuminata banksia* (from Sardos et al., 2016), **(I)** *Solanum muricatum* (courtesy of J. Prohens), **(L)** *Solanum lycopersicum* (seeded and *pat-2* fruits in the genetic background of cv Super Marmande, authors' archive).

The survey confirmed the previous observation that parthenocarpy is taxonomically widespread, being “not uncommon” (Gustafson, 1942) in species producing fruits with several to many seeds while representing a less frequent event in species having monospermic fruits (Figure 3; Roth, 1977). In addition, parthenocarpy was observed mostly among dicot taxa in both the wild and cultivated categories (Table S1). Polyploidy occurred with high frequency among parthenocarpic species. The wide occurrence of parthenocarpy in fruit-crops (65%) is likely the result of a selective pressure for seedlessness during their domestication and breeding. Reasons for such a selection can be several: parthenocarpy (i) releases fruit set from environmental constraints, (ii) may be advantageous for fruit processing, (iii) may improve fruit quality, or (iv) simply represents a feature appreciated by consumers. Selected varieties of watermelons, grapes, *Citrus*, pineapples and bananas are clear examples of fruit-crops where seedlessness is frequent (Varoquaux et al., 2000).

### The Mission of a Seedless Fruit: An Adaptive Role of Parthenocarpy

If parthenocarpy in fruit crops evidently benefited of human selection, the production of seedless fruits in wild or non-fruit crop species (Table S1; Figures 1E,F) represents an apparent biological paradox because they do not directly contribute to the production of offspring. The persistence of parthenocarpy in such species suggests the possibility of adaptive reasons for retaining empty fruits. In monospermic plants, such a role has been based on different mechanisms by which parthenocarpic fruits would reduce seed predation. In this sense, a functional role of seedless fruits has been proposed for wild parsnip (*Pastinaca sativa* L.; Figure 3A), where their occurrence has been related to a defensive value against the parsnip webworms (Zangerl et al., 1991). Given a choice between parthenocarpic and normal fruits, the webworm prefers seedless fruits because of the lower concentration of the deterrent furanocoumarins they contain. In terebinth (*Pistacia terebinthus* L.), parthenocarpy appears to reduce seed predation because predators cannot discriminate between seeded and seedless (deceptive) fruits, as ovaries are not yet enlarged at the time of oviposition; the larvae soon die because in the parthenocarpic fruits there is no endosperm available for feeding (Traveset, 1993). A similar hypothesis has been extended to *Pistacia lentiscus* L. (Verdù and García-Fayos, 1998) and *Bursera morelensis* L. (Ramos-Ordoñez et al., 2008) and even to explain the occurrence of empty seeds in the gymnosperm *Juniperus osteosperma* (Torr.) Little (Fuentes and Schupp, 1998).

The reason given for an adaptive role of parthenocarpy in monospermic species more difficultly applies to plurispermic taxa, where still wild species and non-fruit crops with parthenocarpic fruits have been reported. It is thought that plants have evolved flowers with a great number of ovules as a response to habitats where pollination is more uncertain (Verdù and García-Fayos, 1998). In these cases, a plant with many ovules per flower often experiences a very variable seed/ovule ratio (Burd, 1994). It is well-known that seeds supply the ovary with the hormones necessary for triggering fruit set and development (Sotelo-Silveira et al., 2014); in fact, fruits grow more in portions were seeds are developing (Crane, 1964). Differences in the number of seeds per fruit alter the cost of the fruit for the mother plant: plants invest fewer resources per seed in multi-seeded fruits than in few-seeded fruits (Obeso, 2002). Accordingly, as a strategy to optimize resources, mother plants avoid the development of plurispermic fruits with few seeds (Obeso, 2002). In the case of low pollination rates, the few seeds set are presumable not enough to support fruit growth, thus causing abscission. Under these circumstances, parthenocarpic capacities could offer the opportunity to accomplish fruit development and the production and dispersal of the few seeds that otherwise would be lost. The high incidence of parthenocarpy in plants with separate sexes (Figures 3E,F), that experience low pollination rates more often than hermaphrodites, supports this suggestion. In this scenario, genes for (facultative) parthenocarpy provide an adaptive advantage which would lead to a boost of seed development-related hormones (auxin and gibberellins) and the chance to produce few seeds for the next generation. In a parallel example, it was demonstrated that reduced reproductive output resulting from early flowering offers an advantage in the adaptation of invasive weeds to higher latitudes (Kralemann et al., 2018).

### Inventory of Parthenocarpic Sources in Tomato

The tomato is an important vegetable crop worldwide and a model for the study of fruit set and development (Foolad, 2007). Harnessing parthenocarpy in this species has been an important breeding objective to uncouple fruit set from environmental constrains and to provide quality traits such as higher soluble solids and ascorbic acid content (Figure 3L; Gorguet et al., 2005; Mazzucato and Picarella, unpublished results).

Although recent reviews have generally focussed on describing reverse genetics experiments (Gorguet et al., 2005; Shinozaki and Ezura, 2016; Joldersma and Liu, 2018), in the past inventories have been compiled that provide information on the origin and use of parthenocarpic accessions obtained by mutagenesis and conventional breeding (Philouze, 1983; George et al., 1984; Lukyanenko, 1991).

With possibly the only exception of the *parthenocarpic fruit* (*pat*) mutant obtained by EMS treatment (Bianchi and Soressi, 1969), all the parthenocarpic lines described were derived from crosses involving the cultivated tomato and different relative species (Table 1). *pat-2*, one of the best studied locus for parthenocarpy in tomato, was first described by Dovedar (1973, cited by Philouze, 1983) in the progeny of a cross involving *S. habrochaites* S. Knapp & D. M. Spooner (formerly *Lycopersicon hirsutum* Dunal). *pat-k* was retrieved in the progeny of a cross involving *pat-2*, although it segregated as an independent locus (Takisawa et al., 2017). Two parthenocarpic lines obtained in The Netherlands (Zijlstra, 1985) were subsequently classified in the *pat* series; IVT-1 was related to a digenic control (*pat-8*/*pat-9*), whereas IVT-2 was referred to as *pat-5* (Gorguet et al., 2008). Both these lines were derived from crosses with different wild species (Table 1). Also IL5-1 (*pat-6*/*pat-7*) was obtained after a cross with *S. habrochaites* (Gorguet et al., 2008).

**Table 1:** Sources of genetic parthenocarpy in tomato described in the literature (species are reported with taxonomic names adopted after Peralta et al., 2008).

**Line name**	**Mutation/s**	**Cultivated parent**	**Wild/wide-related parent**	**References**
*Stock 2524*	*pat*	Cv Ventura	–^a^	Bianchi and Soressi, 1969
Severianin	*pat-2*	Byzon	*S. habrochaites*	Dovedar, 1973 (cited by Philouze, 1983) (Philouze and Maisonneuve, 1978)
Kyo-temari	*pat-k*	Severianin	–^b^	Takisawa et al., 2017
RP 75–59	*pat-3*/*pat-4*	Atom	Bubjekosoko ^c^	Reimann-Philipp, 1968 (cited by Philouze, 1983)
IVT-2	*pat-5*	*S. lycopersicum*	*S. peruvianum*	Zijlstra, 1985
IL5-1	*pat-6*/*pat-7*	*S. lycopersicum*	*S. habrochaites*	Gorguet et al., 2008
IVT-1	*pat-8*/*pat-9*	*S. lycopersicum*	*S. habrochaites*	Zijlstra, 1985; Gorguet et al., 2008
–	–	Bonny best	Large cherry	Hawthorn, 1937
*Pridneprovskij*	–	*S. lycopersicum*	*S. lycopersicum* var. *cerasiforme*	Kraevoj, 1949 (cited by Philouze, 1983)
–	–	*S. lycopersicum*	*S. peruvianum*	Lesley and Lesley, 1953
*PI190256*	–	New Caledonia	*S. lycopersicum* var. *cerasiforme*	Johnson and Hall, 1954
*Hybrids n. 1641,1154*	–	*S. lycopersicum*	*Cyphomandra* spp	Luneva, 1957 (cited by Philouze, 1983)
*Lyconorma, Lycoprea*	–	*Priora*	*Heinemänns Jubilaum* ^d^	Reimann-Philipp, 1977 personal communication (cited by Philouze, 1983)
*Oregon cherry*	–	*Farthest North*	*S. habrochaites, L. pimpinellifolium*	Baggett and Frazier, 1978a
*Oregon T 5-4*	–	*Farthest North*	*S. habrochaites*	Baggett and Frazier, 1978b
*Oregon 11, Gold Nugget*	–	*S. lycopersicum*	*S. pimpinellifolium, S. habrochaites*	Baggett and Frazier, 1982
–	–	*S. lycopersicum*	*S. neorickii*	Philouze, unpublished (cited by Philouze, 1983)
*P26, P31*	–	*S. lycopersicum*	*S. pennellii*	Stoeva et al., 1985 (cited by Lukyanenko, 1991)
Carobeta	–	*S. lycopersicum*	*S. pimpinellifolium*	Georgiev and Mikhailov, 1985
*RG*	–	*S. lycopersicum*	*S. cheesmaniae* var. *minor*	Mikhailov and Georgiev, 1987 (cited by Lukyanenko, 1991)

a *Not determined, not applicable*.

b *Derived from a cross between a variant from “Severianin” and a non-parthenocarpic cultivar*.

c *Classified in the IPK seedbank (http://www.ipk-gatersleben.de/genbank/) as a L. esculentum Mill. convar. parvibaccatum Lehm. var. cerasiforme (Dunal) Alef*.

d *Classified in the IPK genebank as L. esculentum Mill. convar. fruticosum Lehm. var. pygmaeum Lehm*.

The same involvement of wide crosses is found in the pedigree of those sources of parthenocarpy that were not genetically characterized. A contribution from *S. lycopersicum* var. *cerasiforme* (formerly *L. esculentum* var. *cerasiforme)* is traced in the first report of parthenocarpic fruits in tomato (Hawthorn, 1937), in the Ukrainian selection Pridneprovskij (Kraevoj, 1949, cited by Philouze, 1983), in PI190256 (Johnson and Hall, 1954), and possibly in the pedigree of the varieties Lyconorma and Lycoprea whit the parental accession Heinemänns Jubileum (Reimann-Philipp, 1977 personal communication cited by Philouze, 1983). In addition, line RP 75-59 derived from a cross between Atom and Bubjekosoko, British and Russian cultivars, respectively, was characterized as *pat-3*/*pat-4* (Reimann-Philipp, 1968, cited by Philouze, 1983). Bubjekosoko is a cherry tomato type (Mahmoud and El-Eslamboly, 2014), classified in the IPK seedbank (http://www.ipk-gatersleben.de/genbank/) as a *L. esculentum* Mill. convar. *parvibaccatum* Lehm. var. *cerasiforme* (Dunal) Alef taxon.

Several parthenocarpic selections obtained in Oregon had *S. pimpinellifolium* and *S. habrochaites* in their pedigree (Baggett and Frazier, 1978a,1978b, 1982). *S. pimpinellifolium* was also a relative of Carobeta, a variety carrying the introgression of the *B* allele responsible for the orange fruit color due to high content of β-carotene (Georgiev and Mikhailov, 1985).

Facultative parthenocarpy was also found after more distant crosses of the cultivated tomato with *S. cheesmaniae* (L. Riley) Fosberg (formerly *L. cheesmaniae* L. Riley; Mikhailov and Georgiev, 1987, cited by Lukyanenko, 1991), *S. neorickii* D.M. Spooner et al. (formerly *L. parviflorum* C.M. Rick et al.; Philouze, 1983), *S. pennellii* Correll [formerly *L. pennellii* (Correl) D'Arcy; Stoeva et al., 1985, cited by Lukyanenko, 1991], *S. sitiens* I.M. Johnst. [formerly *L. sitiens* (I.M. Johnst.) J.M.H. Shaw; R. Chetelat, personal communication], *S. peruvianum* L. [formerly *L. peruvianum* (L.) Miller; (Lesley and Lesley, 1953)] and *Cyphomandra* spp. (Luneva, 1957 cited by Philouze, 1983).

The association of parthenocarpy and wide hybridizations was first addressed by Lesley and Lesley (1953), who attributed the phenotype to an “exceptional combination of genes coming from the two species that involved an excessive production of auxin.” After that, this association was mentioned (Philouze, 1983; Ho and Hewitt, 1986), but no specific hypothesis as to the mechanism of this observation was proposed.

### Wide Hybridization as a Force Driving Departures From Normal Sexual Plant Reproduction

As all developmental processes, sexual plant reproduction is a complex pathway depending on external and internal stimuli and regulated by multi-dimensional checkpoints and interactions. However, early studies underlined the modular and hierarchical structure of reproductive development algorithms in plants (Haig, 1990). This suggestion was developed by modern synthetic biologists that support the awareness that cells and organisms are organized as a hierarchical combination of functional modules (Benner and Sismour, 2005). Following the extensive amount of data produced by high-throughput sequencing methods, the modular organization of cellular systems has emerged and led to the notion that they could be treated similarly to traditional engineering systems (electrical or mechanical). It seems therefore possible to use novel combinations of existing modules to achieve new functions in a given organism in a predictable way (Cameron et al., 2014).

First inventories of species showing agamospermic behavior revealed that apomixis occurs almost exclusively in taxa characterized by hybrid origin and polyploidy (Asker and Jerling, 1992). Following this evidence, Carman (1997) elaborated the “duplicate-gene asynchrony hypothesis” for the genetic control of apomixis. The theory, also known as “no-gene theory,” postulates that modular sets of genes inherited from different species may manifest asynchronous expression in terms of heterochronicity (wrong expression or asynchrony in time) and/or heterotopicity (wrong expression in space) and as such explain modifications of the reproductive system like apospory, diplospory, and apomixis as a whole (Carman, 1997). Thus, apomixis and related reproductive variations would result from developmental programs that are ectopically and/or prematurely expressed due to the misregulation of duplicate genes in polyploids, mesopolyploids, or paleopolyploids (Carman, 1997).

Accordingly with Carman's hypothesis, the so called “stages of evolution” of apomixis begin with weak facultative expression that has been consolidated by mutations. This is corroborated by the fact that “tendencies toward apomixis” are common in natural and synthetic polyploids (Asker and Jerling, 1992; Osborn et al., 2003). Interestingly, according to the “fading borders model,” gradual heterotopic variation in the level of expression of floral organ identity genes resulted in the evolution of floral organ morphology across diversification of angiosperms, from the basalmost to the more evolved lineages (Soltis et al., 2007).

Experimental evidence of the “no-gene” theory has recently emerged from analysis of transcriptomes in apomicts. The occurrence of heterochronic gene expression, compared to sexual types, has been experimentally displayed in the diplosporous *Tripsacum dactyloides* (L.) L. (Grimanelli et al., 2003; Bradley et al., 2007) and *Boechera retrofracta* (Graham) Á. Löve & D. Löve (Sharbel et al., 2010).

### Parthenocarpy as a Consequence of Wide Hybridization

Examining the common origin from interspecific crosses in tomato sources for parthenocarpy leads to postulate a similar “no-gene” (that means “no-mutation”) genetic basis also for parthenocarpy. When different genomes are “colliding” (sensu Carman, 2001) after interspecific or intraspecific wide crosses, modification of developmental programs controlling fruit set may occur by overlapping of regulatory signals that may be spatial-temporally asynchronous and thus drive the development of the ovary independently of fertilization. Such a modification can eventually become fixed in populations if adaptive advantages with the new developmental program exist as it is found in apomictic plants and parthenocarpic crops.

From an evolutionary point of view, gene interactions are postulated to be functional within species, but incompatible or deleterious in hybrids (Muller, 1942). Hybrid lethality may therefore function as a driver of seed abortion that can lead to stenospermocarpy. However, reunification of divergent genomes may more simply lead to novel patterns of expression in target loci and genetic or epigenetic changes resulting in altered gene expression, gene silencing, novel tissue specificity or activation of transposable elements (Comai et al., 2000). That these events may lead to improved fitness is witnessed by the common hybrid origin of invasive plants (Blair and Hufbauer, 2010) and by the attitude of apomicts to colonize disturbed habitats (“geographical parthenogenesis”; Cosendai and Hörandl, 2010).

Related, sexually compatible species, may present different time spans for reproductive developmental modules such as development of sporangia, meiosis, gametogenesis, fertilization, and fruit set. A hybrid between these taxa could inherit different modules that may not be synchronized as in the parents. Although, it is known that a specific set of genes is activated exclusively after pollination/fertilization (Vriezen et al., 2008; Ruiu et al., 2015), it is also recognized that fruit set may be driven by fertilization-independent pathways, activation of downstream genes or removal of repressors driven by mutations or hormone treatments (Pascual et al., 2009; Wang et al., 2009; Ruan et al., 2012). In this scenario, the effect of asynchrony in hybrid gene expression may be crucial to induce fruit set positive signals before fertilization can take place.

This hypothesis is supported by a number of observations that have emerged from studies on genetic parthenocarpy in tomato as detailed below:

- (1) An oligogenic control of the trait has been reported in a number of cases (*pat-3*/*pat-4, pat-6*/*pat-7*, and others, Table 1). Also in the *pat-2* mutant, although the phenotype has been shown to depend on a single mutated gene (Nunome et al., 2013), its penetrance is dependent on the presence of the minor modifier gene *mp* (Vardy et al., 1989). This indicates that the trait is often the result of a combination of more than one genetic determinant.
- (2) Parthenocarpy may occur in association with abnormal development of male or female floral organs. Defects in early ovule development have been associated with parthenocarpy in the variety Pridneprovskij (Ludnikova, 1970, cited by Philouze, 1983) and in the *pat* (Mazzucato et al., 1998) and *pat-k* (Takisawa et al., 2017) mutants in tomato and in pepper parthenocarpic lines (Tiwari et al., 2011). This indicates that in specific cases parthenocarpy occurs as a downstream or combined effect of alterations that are expressed early to module organ development and identity. Ultimately such alterations may affect later processes like ovary growth.
- (3) Often, strong parthenocarpy arose from parents without or just with weak tendencies to seedlessness. In Poland, the cross of two Canadian varieties, “Early North” (with limited parthenocarpic attitude) and “Beaverlodge 6703” (with no parthenocarpy), resulted in tomato lines with strong parthenocarpic capacity in conditions unfavorable to fertilization (Kubicki and Michalska, 1978).
- (4) Evidence of parthenocarpy often appears in the segregation of F_1_ hybrids (G.P. Soressi, personal communication) that are obtained from crosses between distantly related genotypes including conspicuous introgressions of wild germplasm (Lin et al., 2014).
- (5) Finally, in species other than tomato genomic perturbations such as changes in ploidy have been reported as the basis for parthenocarpy; the link between triploidy and parthenocarpy is established (and exploited) in species like watermelon and banana (Kihara, 1951; Ortiz and Vuylsteke, 1995; Varoquaux et al., 2000). Often, triploidy is achieved by interspecific crosses as in pummelo (*C. grandis* x *C. paradisi*) and other *Citrus* hybrids (Soost and Cameron, 1985; Vardi et al., 2008).

All these observations support the idea that the expression of parthenocarpy in many tomato lines is the consequence of particular combinations of (sets of) genes involved in reproduction, more than that of a single gene that underwent spontaneous or induced mutation. This possibility is supported by the high occurrence of polyploidy among parthenocarpic species that has been described and discussed before (Figure 2).

### Transcriptomics of Tomato Fruit Set Supports the Hybrid Origin of Parthenocarpy

A number of studies have focussed on the transcriptomic description of pollination-dependent and pollination-independent fruit set in tomato, comparing systems where parthenocarpy was driven by hormone treatment (Vriezen et al., 2008; Tang et al., 2015), expression of inductive genes (Martinelli et al., 2009; Molesini et al., 2009) or silencing of repressors (Wang et al., 2009; Mounet et al., 2012). Genetic parthenocarpy has been investigated at the transcriptomic level only in the *pat3*/*pat4* (Pascual et al., 2009) and in the *pat* (Ruiu et al., 2015) mutants, but the former was the only system analyzed where seedlessness was obtained after hybridization. In this study, the authors concluded that the stage of anthesis was the most different between the wild-type and the *pat3*/*pat4* parthenocarpic line and the key point at which many genes are differentially expressed. However, normal and parthenocarpic fruit set were transcriptionally similar, without drastic changes in gene expression between the two genotypes (Pascual et al., 2009). Thus, transcriptomic analysis of fruit set in *pat3*/*pat4* suggested the importance of differential gene expression in time, although this study could not explicitly conclude that heterochronicity was the driving force of the entire process.

Transcriptomic studies at the fruit set stage have also been carried out in tomato wild relatives (Pattison et al., 2015; Dai et al., 2017). However, due to the lack of parallel studies, the available databases offer scarce possibility to evaluate heterochronicity in gene expression between wild and cultivated forms. To get insights into the degree of correlation of gene expression in cultivated and wild forms, genes involved in fruit set were selected from the analysis on the *pat* mutant (Ruiu et al., 2015) addressing those transcripts that are up-regulated after anthesis in the WT but not in the mutant (Pollination-dependent genes, PD group) and those that are up-regulated after anthesis in the mutant but not in the WT (Fruit growth genes, FG group). For all these genes, the logFC between anthesis and 4/5 DPA was calculated from expression data retrieved in the TEA database in M82 and *S. pimpinellifolium*, respectively and separately for different ovary tissues (pericarp, placenta, septum).

**Figure 4:**
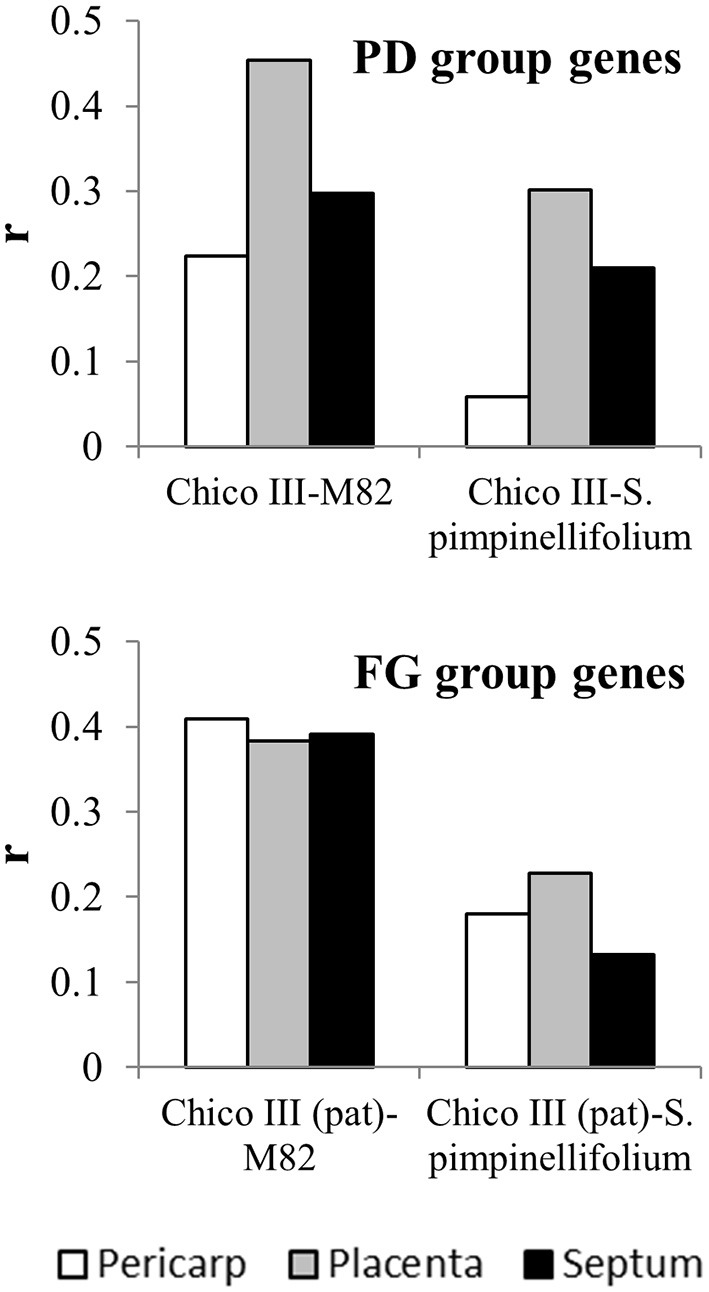
Correlation coefficients between expression of fruit set-related genes in cultivated and wild forms of tomato. logFC of the expression few days after anthesis and at anthesis of genes up-regulated by pollination (PD group) and early fruit growth (FG group) in cv Chico III ovaries (Ruiu et al., 2015) and in M82 and *S. pimpinellifolium* pericarp, placenta and septum (Tomato Expression Atlas at the Sol Genomics Network website, sgn, https://solgenomics.net;Shinozaki et al., 2018).

The correlations found between the two cultivated forms (Chico III and M82) ranged from 0.22 to 0.45, being more differentiated among tissues in the PD than in the FG gene group (Figure 4). All the correlations between Chico III and *S. pimpinellifolium* showed lower values, with a decrement that ranged between 30 and 74% in the PD group and between 39 and 66% in the FG gene group (Figure 4). Making allowance of the differences in the experimental systems compared, this analysis provided an indirect indication that specific sets of genes are differentially activated at the fruit set interface between cultivated and wild tomato.

### Further Commonalities Between Apomixis and Parthenocarpy

A similar mechanistic basis for apomixis and parthenocarpy may also be deduced by the fact that the two phenomena seldom occur in the same taxon, as reported in birch (Bogdanov and Stukov, 1976), in subtropical species of the Asteraceae (Werpachowski et al., 2004), in *Citrus* (Vardi et al., 2008) and in *Musa* (Okoro et al., 2011).

Koltunow et al. (2002) treated apomixis and parthenocarpy as phenomena with possible common bases by highlighting a number of commonalities between the two processes. First, they both derive from the disruption of molecular mechanisms that prevent the development of a floral organ (ovule and carpel, respectively) in the absence of fertilization. As such, the ovule becomes a fundamental structure in the molecular signaling underlying these mechanisms. Moreover, the two processes are stochastic and both characterized by facultativeness, that makes possible the coexistence of modified and normal processes within the same individual (Koltunow et al., 2002).

A further common element is the involvement of B-class MADS-box homeotic transcription factors in both apomixis and parthenocarpy. The fact is paradoxical since, according to the ABC model for floral organ formation, B-class genes are typically expressed in the second and third floral whorl and contribute to the identity and development of petals and stamens (Weigel and Meyerowitz, 1994). However, several authors reported the expression of *SlDEF* [the tomato ortholog of *DEFICIENS* (*DEF*) in *Antirrhinum majus* and of *APETALA3* (*AP3*) in *Arabidopsis thaliana*] in the fourth floral whorl (Mazzucato et al., 2008; Tang et al., 2015). In the aposporous apomict *Hieracium piloselloides* Vill., the ovule presents a downregulation of *DEFH* in a broad zone of the chalaza that coincides with the region where aposporous initials differentiate; such a downregulation is not seen in sexual ovules (Guerin et al., 2000). In parallel, differential expression of *DEF* homologs have been reported in ovaries showing wild-type or parthenocarpic behavior. In tomato, *SlDEF* shows a peak of expression in ovaries at anthesis, that coincides with the signal that arrests ovary growth (Vriezen et al., 2008; Wang et al., 2009); such an accumulation is absent in ovaries that develop autonomously in the *pat* mutant (Mazzucato et al., 2008; Ruiu et al., 2015). In parallel with these findings, mutated alleles (apple; Yao et al., 2001) or epialleles (oil palm; Ong-Abdullah et al., 2015) of B-class MADS box genes have been shown to cause parthenocarpy and defects in their expression showed interference with fruit set in grapevine (Fernandez et al., 2013).

## Conclusions

The inventory of angiosperm species showing parthenocarpic behavior and of the sources of parthenocarpy in the specific case of tomato offered novel insights into the role that autonomous ovary development may have played in natural evolution and in the man-driven activity of selection and breeding. The search of novel parthenocarpic species, novel spontaneous and induced mutants as well as novel genes involved in the phenomenon will give support to the models proposed and new insights into the control of ovary development in angiosperms. In parallel with apomixis, such insights will pave the way to new opportunities to harness a modification of the reproductive system in tomato and in other fruit crops that is of great interest to modern breeding.

## Author Contributions

AM and MP contributed conception and design of the study. MP organized the database and wrote the first draft of the manuscript. AM wrote sections of the manuscript. All authors contributed to manuscript revision, read and approved the submitted version.

### Conflict of Interest Statement

The authors declare that the research was conducted in the absence of any commercial or financial relationships that could be construed as a potential conflict of interest.
